# Complete synchronization of the global coupled dynamical network induced by Poisson noises

**DOI:** 10.1371/journal.pone.0188632

**Published:** 2017-12-07

**Authors:** Qing Guo, Fangyi Wan

**Affiliations:** School of Aeronautics, Northwestern Polytechnical University, Xi’an, Shaanxi, China; Lanzhou University of Technology, CHINA

## Abstract

The different Poisson noise-induced complete synchronization of the global coupled dynamical network is investigated. Based on the stability theory of stochastic differential equations driven by Poisson process, we can prove that Poisson noises can induce synchronization and sufficient conditions are established to achieve complete synchronization with probability 1. Furthermore, numerical examples are provided to show the agreement between theoretical and numerical analysis.

## Introduction

Noise-induced synchronization in chaotic systems is an interesting phenomenon due to the fact that multiplicative and/or additive noises are ubiquitous in natural and synthetic systems, and up to now it has been studied by many investigators from different areas [1–3]. Lin and his co-workers [4,5] have presented some sufficient conditions of complete synchronization between two unidirectionally coupled chaotic systems disturbed by Gaussian white noise. Later, Xiao and his co-workers have analyzed the effect of Gaussian white noise in bidirectionally coupled piecewise linear chaotic systems [6, 7] and global coupled dynamical network which consists of many nodes. Cao et al. studied the complete synchronization linear stochastic coupled network and gave the sufficient conditions for complete synchronization of network under adaptive control [8]. It has been found that many real systems should be described by the complex dynamical networks (CDNs). The CDNs widely exist in the areas of the Internet, metabolic pathways, the World Wide Web, food-webs, ecosystems, global economic markets, social networks and neuronal network [9–20].

In previous studies, noises were usually assumed to be Gaussian cases, which have been used to approximate different kinds of stochastic perturbations with/without jumps in many situations. However, Gaussian distributions are not appropriate in some practical situations and environments while there may exist large external and/or internal fluctuations [21–28]. It is well known that in the real world, beside Brown noises, there is a very common but important kind of random noises: Poisson noises. Poisson noises which can model these large external and/or internal fluctuations have been observed in various systems such as storage systems, economic systems, biological systems and so on [29–32]. And it is widely known that Poisson process can be viewed as a sequence of independent identically distributed random pulses with the left limits and right-continuous sample paths. As a consequence, the behaviors of the dynamical systems driven by Poisson noise are very different from the stochastic systems driven by Gaussian white noise [33–40]. Thus, it is very important to investigate the dynamic behaviors, such as the synchronization phenomena, for complex networks perturbed by the Poisson process.

Inspired by above analysis, in this paper, we use the stability theory of stochastic differential equation (SDE) driven by Poisson process to analyze the effect of Poisson noise in the global coupled dynamical network.

## Preparations

*C*_1_(*R*^*k*^) - the space of the functions which have continuous first partial derivatives on *R*^*k*^.
*C*_*b*_(*R*^*k*^) - the space of bounded continuous functions on *R*^*k*^.
$C_{b}^{1}\left(R^{k}\right)$ -the subspace of *C*_*b*_(*R*^*k*^) constituted by the functions have continuous first partial derivatives.
$C_{1}^{0}\left(R^{k}\times R_{+}\right)$ - class of functions *V*(*x*,*t*) which have continuous first partial derivatives on *R*^*k*^ × *R*_+_ except possibly at the point *x* = 0.

### 2.1. Definition

A function *V*(*x*,*t*) is said to be positive definite (in Lyapunov’s sense) in *R*^*k*^ × *R*_+_, *S*_*h*_ = {*x* ∈ *R*^*k*^: |*x*| < *h*, *h* > 0}, if

(C1) *V*(0,*t*) = 0,*t* ∈ *R*_+_,
(C2) *V*(*x*,*t*) ≥ *W*(*x*), *x* ∈ *S*_*h*_, *t* ∈ *R*_+_, where *W*(0) = 0, *x* ≠ 0, *W*(*x*) > 0.

Obviously, it is negative definite if −*V*(*x*,*t*) is positive definite.

### 2.2. Definition

A function *V*(*x*,*t*) is said to be possessed of an infinitesimal upper limit, if

$$\underset{x\to 0}{\lim}\underset{t>0}{\sup}V\left(x,t\right)=0.$$ (1)

### 2.3. Definition

The trivial solution *X*(*t*) ≡ 0 of the differential equation is said to be

(D1) stochastically stable, if for any *t*_0_ ≥ 0, *ε* > 0,

$$\underset{X_{0}\to 0}{\lim}P\left\{\underset{t\geq t_{0}}{\sup}\left|X\left(t,X_{0},t_{0}\right)\right|>\varepsilon \right\}=0.$$ (2)

(D2) global stochastic asymptotically stable, if it is stochastically stable and also for *t*_0_ ≥ 0, *X*_0_ ∈ *R*^*k*^,

$$P\left\{\underset{t\to \infty }{\lim}X\left(t,X_{0},t_{0}\right)=0\right\}=1.$$ (3)

## SDE driven by Poisson process

Consider a SDE of the following form:

$$dX(t)=b(X(t),t)dt+\sigma (X(t-),t)dP\left(t\right),X(t_{0})=X_{0},$$ (4)

where *b*(*x*,*t*): *R*^*k*^ × *R*_+_ → *R*^*k*^; *σ*(*x*,*t*): *R*^*k*^ × *R*_+_ → *R*^*k*^ × *R*^*m*^, *X*(*t*) is a process with values in *R*^*k*^, the coefficients *b*(*x*,*t*) and *σ*(*x*,*t*) are Borel measurable functions. *P*(*t*) is a m-dimensional Poisson process with parameter *λ* = (*λ*_1_,*λ*_2_,…,*λ*_*m*_)^*T*^ and *P*(0) = 0 with probability 1.

Now, we introduce the Ito’s formula of the SDE with Poisson process [33–35]:

Let $v_{i}^{m}$ be a *m*-dimensional vector with 1 in the *i* th component and zeros elsewhere and {*X*(*t*)} is the solution process of Eq (1), then ∀*V* ∈ *C*_1_(*R*^*k*^ × *R*_+_),

$$\begin{array}{l} V\left(X\left(t\right),t\right)=V\left(X\left(s\right),s\right)+\int_{s}^{t}\frac{\partial V\left(X\left(u\right),u\right)}{\partial u}du+\sum_{i=1}^{k}\int_{s}^{t}\frac{\partial V\left(X\left(u\right),u\right)}{\partial x_{i}}b_{i}(X\left(u\right),u)du\\ \;+\sum_{i=1}^{m}\int_{s}^{t}\left[V\left(X\left(u-\right)+\sigma \left(X\left(u-\right),u\right)v_{i}^{m},u\right)-V\left(X\left(u-\right),u\right)\right]dP_{i}\left(u\right). \end{array}$$ (5)

Let $\tilde{A}$ be the extended weak infinitesimal operator of the process {*X*(*t*,*X*_0_,*t*_0_)}, and $\forall V\in C_{b}^{1}\left(R^{k}\times R_{+}\right)$, we define the operator D:

$$\begin{array}{l} DV\left(x,t\right)≅\frac{\partial V\left(x,t\right)}{\partial t}+\sum_{i=1}^{k}b_{i}\left(x,t\right)\frac{\partial V\left(x,t\right)}{\partial x_{i}}\\ \;+\sum_{i=1}^{m}\lambda_{i}\left[V\left(x+\sigma \left(x,t\right)v_{i}^{m},t\right)-V\left(x,t\right)\right], \end{array}$$ (6)

then $\tilde{A}=D$ [33–35].

Now, we introduce the **Global stochastic asymptotic stability theorem**:

Suppose that there exists a function *V*(*x*,*t*) which satisfies the following conditions:

(C6) $\forall V\left(x,t\right)\in C_{1}^{0}\left(R^{k}\times R_{+}\right)$ is a positive definite function with an infinitesimal upper limit,
(C7) $DV\left(x,t\right),\left(x,t\right)\in R^{k}\times R_{+}$ is a negative definite function.

Then, the solution to Eq (4) is global stochastic asymptotically stable [35].

## Noise induces complete synchronization

Consider the global coupled dynamical network as follows:

$${\dot{x}}_{i}=f\left(x_{i}\right)+c\sum_{i=1}^{n}a_{ij}x_{j},i=1,2,\ldots n.$$ (7)

*x*_*i*_ = (*x*_*i*1_,*x*_*i*2_…..*x*_*in*_)^*T*^ ∈ *R*^*n*^(*i* = 1,2) are state vectors, *f* = (*f*_1_,*f*_2_,*f*_3_……*f*_*n*_)^*T*^: *R*_*n*_ → *R*_*n*_ is a nonlinear function describing the dynamic of an isolated node, and *c* is a positive constant which describes the coupling strength. Here, we consider the global coupled dynamical network with *a*_*ii*_ = −(*n*−1) and *a*_*ii*_ = 1 (*i* ≠ *j*). In fact, the chaotic systems are usually disturbed by noise. Therefore, for system (7), we consider the following model:

$${\dot{x}}_{i}=f\left(x_{i}\right)+c\sum_{i=1}^{n}a_{ij}x_{j}+d_{i}\xi_{i}\left(t\right)\sum_{i=1}^{n}a_{ij}x_{j},i=1,2,\ldots n$$ (8)

where positive constant *d*_*i*_ is the noise strength and *ξ*_1_,*ξ*_2_…*ξ*_*n*_ are Poisson noises.

Moreover, in order to achieve the theoretical result, we also require the function *f* satisfies the following assumption:

### Assumption 1

For any *x* = (*x*_1_,*x*_2_…..*x*_*n*_)^*T*^ ∈ *R*^*n*^, *y* = (*y*_1_,*y*_2_…..*y*_*n*_)^*T*^ ∈ *R*^*n*^, there exists a positive constant *l* satisfying

$${\left(x-y\right)}^{T}\left[f\left(x,t\right)-f(y,t)\right]\leq l{\left(x-y\right)}^{T}(x-y).$$ (9)

Assumption 1 is usually called global Lipschitz condition, and *l* is called Lipschitz constant. For the continuous smooth chaotic systems, it is difficult to find constant *l*, such as Rössler system. However, we can find the constant *l* by inequality proof for some well-known piecewise linear chaotic systems, such as the famous Chua’s circuits [41], the cellular neural network (CNN) neural model [42], and so on.

Actually, the global coupled dynamical network (8) are said to achieve complete synchronization, if *x*_1_ = *x*_2_ = ⋯ = *x*_*n*_ → *m*(*t*), as *t* → ∞. Here, *m*(*t*) ∈ *R*^*n*^ is called as synchronization manifold and satisfies $\dot{m}=f(m)$. However in this paper, we use the $M\left(t\right)=\sum_{i=1}^{n}\frac{x_{i}}{n}$ instead of synchronization manifold *m*(*t*) [41].

Obviously, *M*(*t*) satisfies the following equation:

$$\dot{M}\left(t\right)=\frac{1}{n}\sum_{i=1}^{n}f\left(x_{i}\right)≜G\left(x\right)$$ (10)

Define the synchronization errors *e*_*i*_(*t*) = *x*_*i*_(*t*) − *M*(*t*)(*i* = 1,2…*n*), then, one has the error dynamics

$${\dot{e}}_{i}=f\left(x_{i}\right)-G\left(x\right)-cne_{i}-nd_{i}\xi_{i}\left(t\right)e_{i}+\sum_{i=1}^{n}d_{i}\xi_{i}\left(t\right)e_{i},$$ (11)

Here one should notice that *e*_*i*_(*t*)(*i* = 1,2…*n*) satisfy the following condition:

$$\sum_{i=1}^{n}e_{i}=0.$$ (12)

The above Eq (11) can be written as a matrix form

$$\dot{E}\left(t\right)=F(x)+cC\left(E\right)+H\left(E\right)\dot{P}\left(t\right).$$ (13)

Here $E\left(t\right)=\left(\begin{array}{l} e_{1}\\ \vdots \\ e_{n} \end{array}\right)$, $F(x)=\left(\begin{array}{l} f\left(x_{1}\right)-G\left(x\right)\\ \vdots \\ f\left(x_{n}\right)-G\left(x\right) \end{array}\right)$, $C\left(E\right)=c\left(\begin{array}{l} -ne_{1}\\ \vdots \\ -ne_{n} \end{array}\right)$, $H\left(E\right)=\left(\begin{array}{cccc} \left(1-n\right)d_{1}e_{1} & d_{2}e_{2} & \ldots & d_{n}e_{n}\\ d_{1}e_{1} & \left(1-n\right)d_{2}e_{2} & \cdots & d_{n}e_{n}\\ \vdots & \vdots & \ddots & \vdots \\ d_{1}e_{1} & d_{2}e_{2} & \cdots & \left(1-n\right)d_{n}e_{n} \end{array}\right)$, and $\dot{P}(t)=\left(\begin{array}{l} \xi_{1}(t)\\ \vdots \\ \xi_{n}(t) \end{array}\right)$.

Due to Assumption 1, and the theory of SDE driven by Poisson process [35], one can easily verify that the error Eq (13) possesses a global unique solution denoted by *E*(*t*,*t*_0_,*E*_0_), for any initial condition. Obviously, *E*(*t*,0,0) ≡ 0 is a trivial solution of error system (13). Moreover, after introducing the synchronization errors *e*_*i*_(*t*)(*i* = 1,2⋯*n*), the complete synchronization problem of coupled systems (8) can be translated into the stability problem of the trivial solution of system (13), i.e. the complete synchronization of coupled system (8) corresponds to $\underset{t\to \infty }{\lim}\left\|E\left(t\right)\right\|=0$ with probability 1. Here, ‖•‖ stands for Euclidean norm.

In what follows, we will give sufficient conditions for the complete synchronization of coupled system (8) with probability 1.

We show that the error Eq (13) can be written as

dE(t)=(F(x)+cC(E))dt+H(E)dP(t). (14)

Now, according to the **Global stochastic asymptotic stability theorem** in Part 3, we choose the positive function

$$V\left(E\right)=\frac{1}{2}\left(E^{T}\left(t\right)E\left(t\right)\right),$$ (15)

where *E*^*T*^(*t*) denotes the transpose of *E*(*t*).

By using the infinitesimal operator of SDE driven by a Poisson process to Eq (15) along with system (14). We have

DV(E(t))=∂V(E(t))∂E(t)[F(x)+cC(E)]+∑i=1nλi[V(E(t)+H(E)vin)−V(E(t))]=(e1Te2T…enT)(f(x1)−f(m(t)),f(x2)−f(m(t))⋯f(xn)−f(m(t)))T+c(e1Te2T…enT)(−ne1,−ne2⋯−nen)T+∑i=1nλi[V(E(t)+H(E)vin)−V(E(t))]=∑i=1neiT[f(xi)−f(m(t))]−nc∑i=1neiTei+12∑i=1nλi[(E(t)+H(E)vin)T(E(t)+H(E)vin)−ET(t)E(t)]≤(l−nc)∑i=1neiTei+12∑i=1nλi[(n2−n)di2−2ndi]eiTei. (16)

Obviously, if the coupling strength *c*, noise strength *d*_*i*_, and constants *l*, *λ*, satisfy the inequality

$$\left(l-nc\right)+\frac{1}{2}\lambda_{i}\left[\left(n^{2}-n\right)d_{i}^{2}-2nd_{i}\right]<0,i=1,2\ldots n,$$ (17)

then due to the **Global stochastic asymptotic stability theorem** in Part 3, the trivial solution of system (13) is global stochastic asymptotically stable, and then the synchronization errors *e*_*i*_(*i* = 1,2) converge to zero as *t* → ∞ with probability 1.

We can find that Poisson noise really has a positive effect on the complete synchronization. In the case of the noise strengths *d*_*i*_ = 0, the network become synchronized when *l* − *nc* < 0. From this we can see that there exists a value, all nodes of the network become synchronized when *nc* exceeds this value. In the case of the coupling strength *c* = 0, i.e., the network is only coupled by the internal noise, the whole network become synchronized as

$$l+\frac{1}{2}\lambda_{i}\left[\left(n^{2}-n\right)d_{i}^{2}-2nd_{i}\right]<0,i=1,2\ldots n.$$ (18)

We can see that the noise really have a positive effect on the synchronization. Moreover, the synchronization can be achieved by adding node number, if the global dynamical network (8) is not synchronized under fixed noise strength and coupling strength *c* ≠ 0.

### Remark 1

In terms of the above analysis, we can easily get that the coupled system (8) can achieve complete synchronization with probability 1, when the noise strengths

$$\begin{array}{l} d_{i}<\frac{n\lambda_{i}+\sqrt{n^{2}\lambda_{i}{}^{2}-2\lambda_{i}\left(n^{2}-n\right)\left(l-nc\right)}}{\lambda_{i}\left(n^{2}-n\right)}\\ \;=\frac{n}{\left(n^{2}-n\right)}+\sqrt{\frac{n^{2}}{{\left(n^{2}-n\right)}^{2}}-\frac{2\left(l-nc\right)}{\lambda_{i}\left(n^{2}-n\right)}}\\ \;\leq \frac{2n}{\left(n^{2}-n\right)}\leq 2, \end{array}$$ (19)

and $\lambda_{i}\geq \frac{2\left(n^{2}-n\right)\left(l-nc\right)}{n^{2}}$.

## Numerical examples

In this section, the example is provided and some numerical simulations are performed to verify the theoretical results. This part, we use Runge-Kutta methods to measure the synchronization, let *n* = 2, *d*_1_ = *d*_2_ = *d* in system (8), and we define the following quantities: error(*t*) = |*x*_1_ − *x*_2_|, and *x*_*i*_ = (*x*_*i*1_,*x*_*i*2_…..*x*_*in*_)^*T*^, *i* = 1,2 represents the solution of Eq (8).

### Example

In this example we use the Chua’s circuits [41] which can be depicted by three-dimensional differential equation

$$\dot{x}=Dx+Tg(x),$$ (20)

where *x* = (*x*_1_,*x*_2_,*x*_3_)^*T*^ ∈ *R*^3^ is the state vector,

$$D=\left(\begin{array}{ccc} -a & a & 0\\ b & -b & c\\ 0 & -h & 0 \end{array}\right),T=\left(\begin{array}{ccc} -a & 0 & 0\\ 0 & 0 & 0\\ 0 & 0 & 0 \end{array}\right),g\left(x\right)={\left(g\left(x_{1}\right),g\left(x_{2}\right),g\left(x_{3}\right)\right)}^{T},$$ (21)

in which *a* = *G*/*C*_1_,*h* = *G*/*C*_2_,*c* = 1/*C*_2_,*d* = 1/*L*,*G* is resistance, *C*_1_,*C*_2_ are capacitors, *L* is the inductor, *x*_1_,*x*_2_ denote the volt-age across *C*_1_,*C*_2_, respectively, *x*_3_ is the current through *L*. In particular, the static nonlinearity of Chua’s diode is the piecewise linear curve given by

$$g(x)=m_{0}x+1/2\left(m_{1}-m_{0}\right)\left(\left|x+B\right|-\left|x-B\right|\right),$$ (22)

where *m*_0_,*m*_1_,*B* are the parameters.

**Fig 1:**
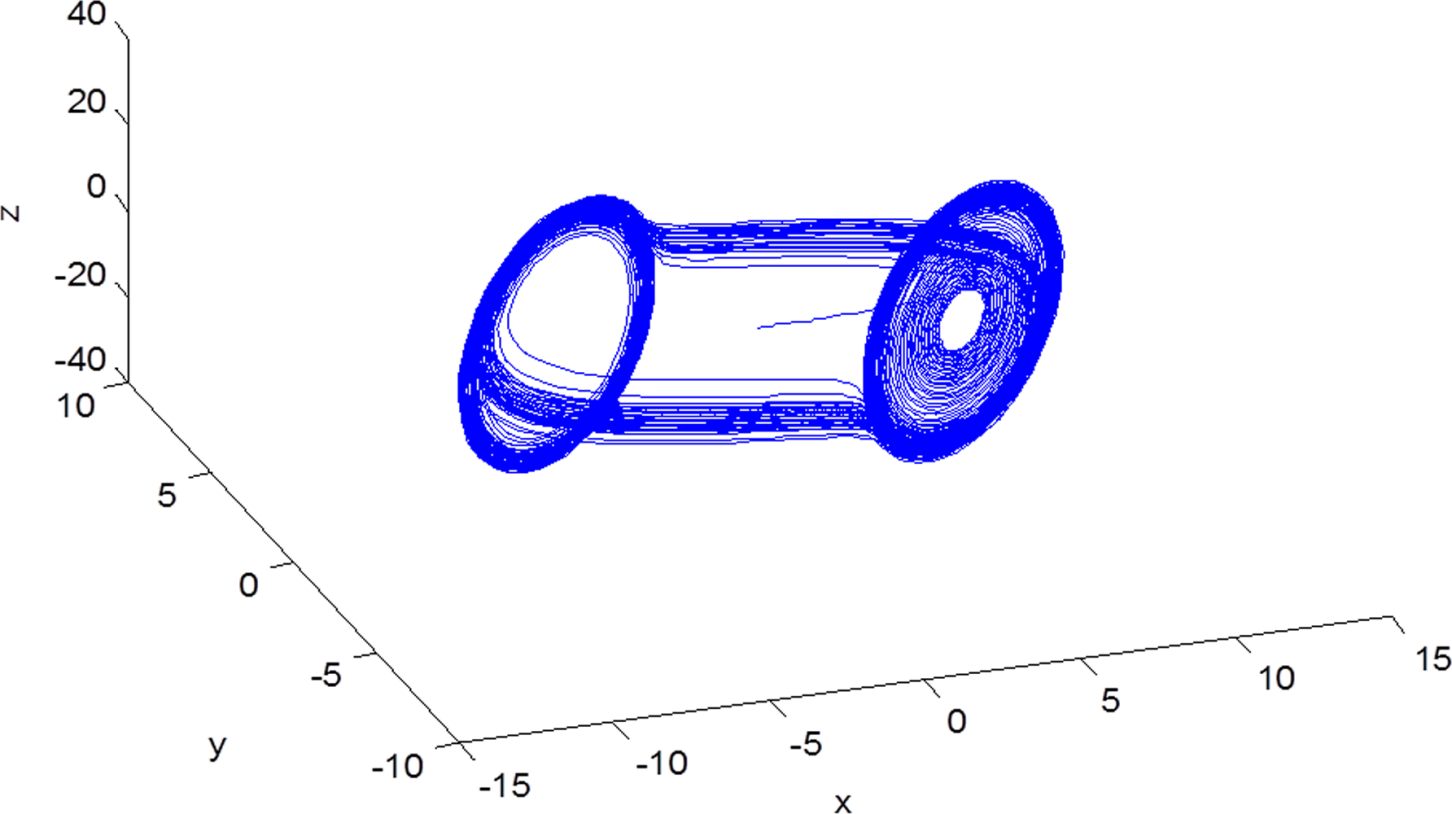
Chaotic attractors generated by the Chua’s circuits (11) after transient time *T* = 1000 has been removed.

On the basis of the Ref. 36, under the above parameters system (20) is chaotic (Fig 1).

We can verify

$$\begin{array}{l} \left|g\left(x_{1}\right)-g\left(y_{1}\right)\right|=\left|m_{0}\left(x_{1}-y_{1}\right)+\frac{1}{2}\left(m_{1}-m_{0}\right)\left[\left(\left|x_{1}+B\right|-\left|x_{1}-B\right|\right)-\left(\left|y_{1}+B\right|-\left|y_{1}-B\right|\right)\right]\right|\\ \;\leq \left(\left|m_{0}\right|+\left|m_{1}-m_{0}\right|\right)\left|x_{1}-y_{1}\right|. \end{array}$$ (23)

For any *x* ∈ *R*^3^, *y* ∈ *R*^3^, we have

$$\begin{array}{l} {\left(x-y\right)}^{T}\left(Dx-Dy+Tg(x)-Tg(y)\right)\leq {\left(x-y\right)}^{T}D\left(x-y\right)+\left|{\left(x-y\right)}^{T}T\left(g(x)-g(y)\right)\right|\\ \;\leq {\left|\left(x-y\right)\right|}^{T}\left[\left(\left|m_{0}\right|+\left|m_{1}-m_{0}\right|\right)\left|T\right|+D\right]\left|\left(x-y\right)\right|\\ \;\leq \lambda_{m}{\left(x-y\right)}^{T}\left(x-y\right). \end{array}$$ (24)

Take the parameters and noise intensity matrices as

$$a=7,b=0.35,c=0.5,d=7,m_{0}=-1/7,m_{1}=-40/7,B=1,$$ (25)

where

$$\left|T\right|=\left(\begin{array}{ccc} 7 & 0 & 0\\ 0 & 0 & 0\\ 0 & 0 & 0 \end{array}\right),D=\left(\begin{array}{ccc} -7 & 7 & 0\\ 0.35 & -0.35 & 0.5\\ 0 & -7 & 0 \end{array}\right),$$

and *λ*_*m*_ ≈ 33.0731 is the maximum eigenvalue of |*T*| − *D*. So we can acquire the constant *l* ≈ 33.0731 in Assumption 1.

By the theoretical results in Sec. 4, *λ*_1_ = 30, *λ*_2_ = 50, the two coupled systems are synchronized with probability 1 with the noise strengths 0.5838 < *d* < 1.416. Therefore, we compute the error(*t*) with different *d* to find the agreement with the theoretical results.

**Fig 2:**
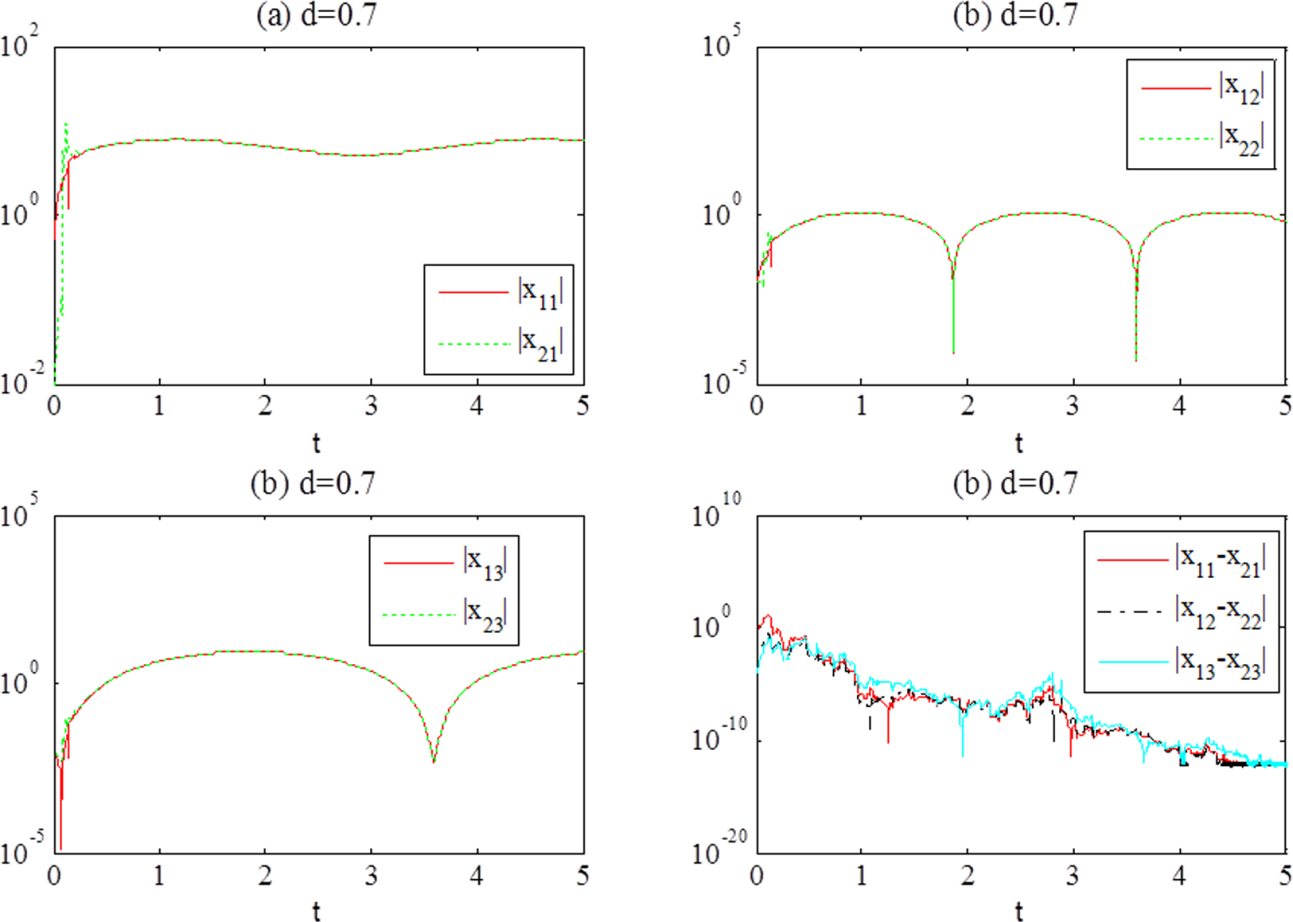
(a-c) Responses of the systems (11) after synchronization achievement, (d) the temporal evolution of error(*t*) with *d* = 0.7.

**Fig 3:**
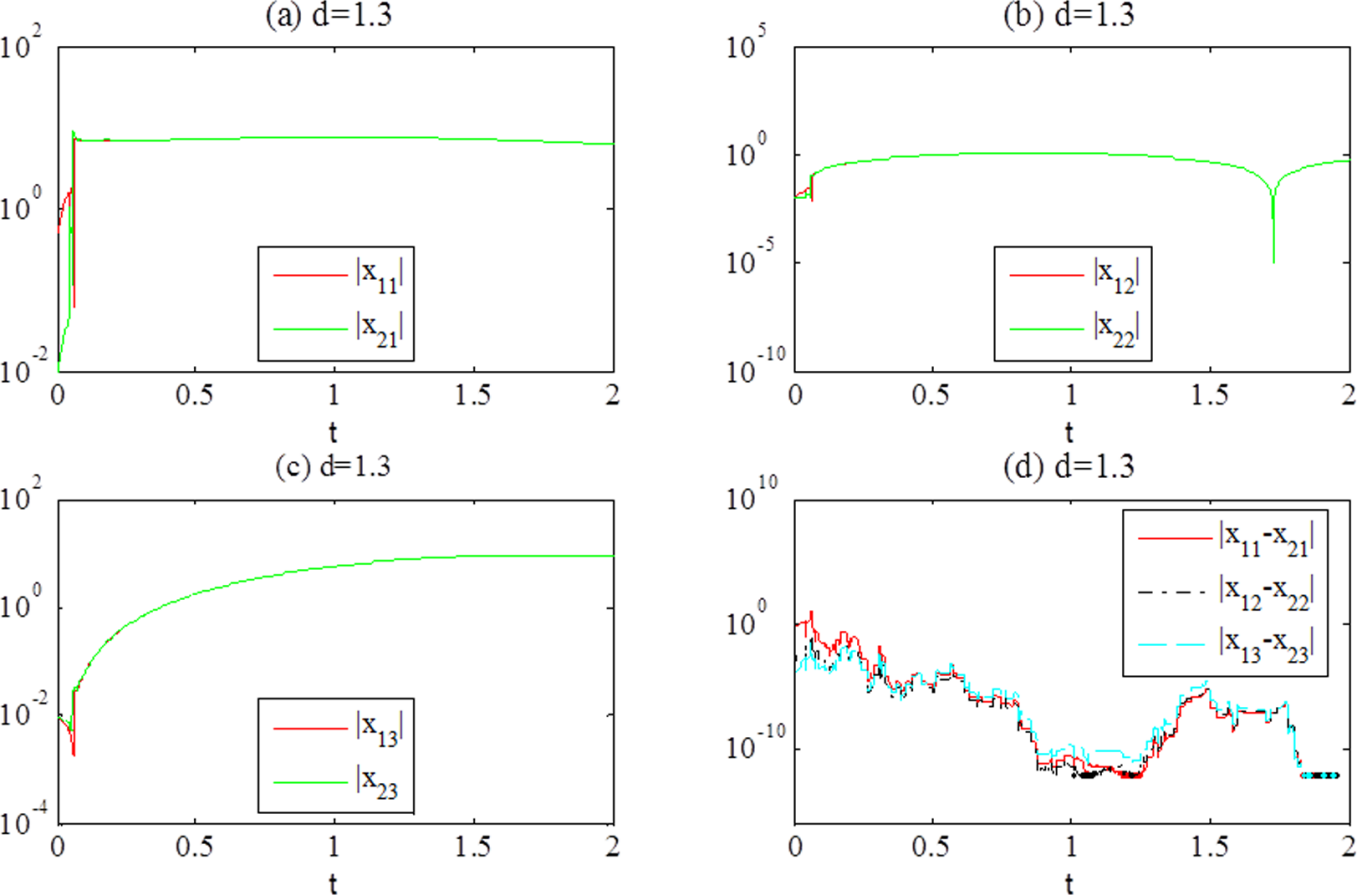
(a-c) Responses of the systems (11) after synchronization achievement, (d) the temporal evolution of error(*t*) with *d* = 1.3.

The simulation results are shown in Fig 2 with *d* = 0.7, and Fig 3 with *d* = 1.3.

**Fig 4:**
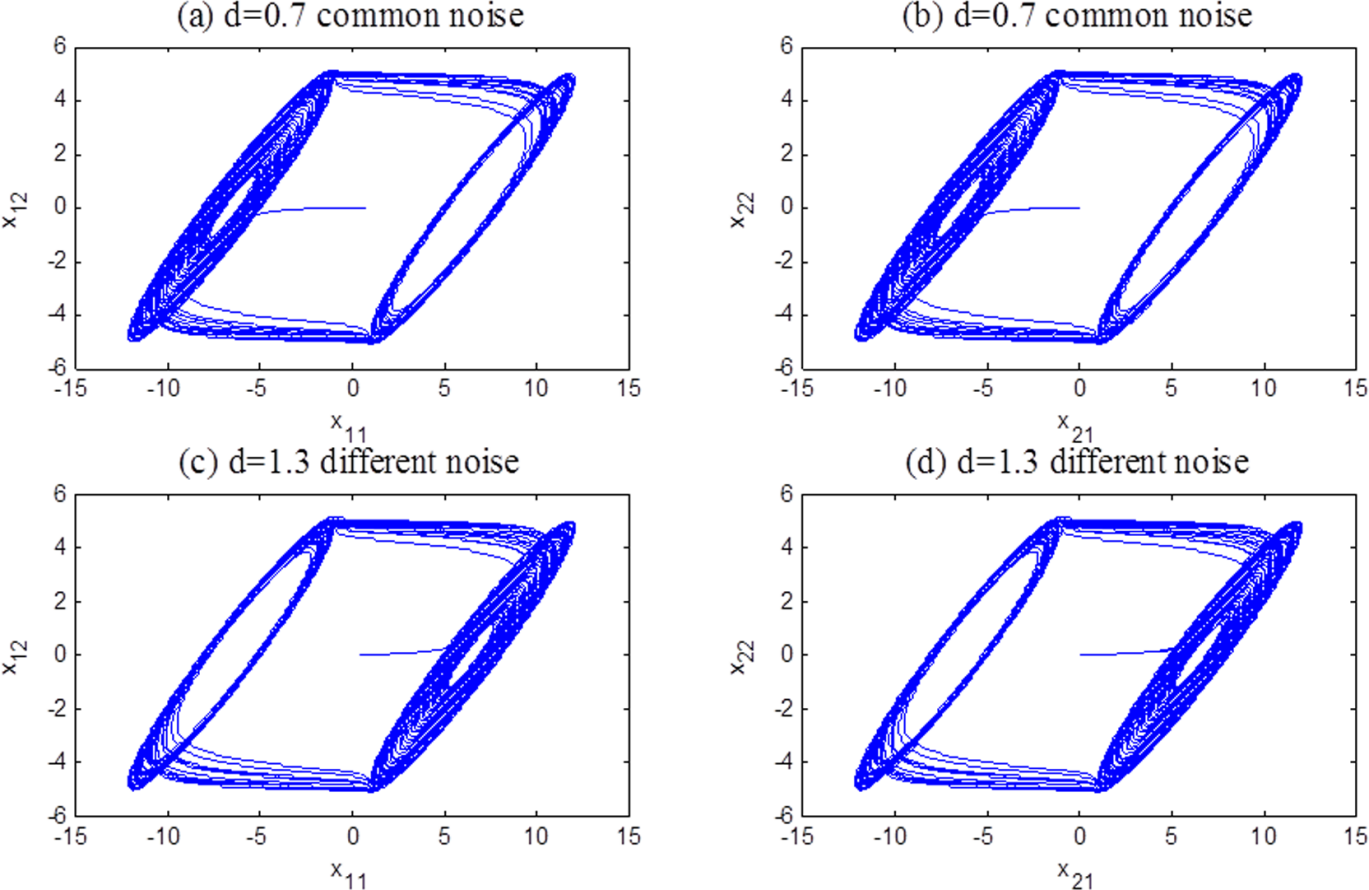
Chaotic attractors of system (11) after synchronization achievement.

In terms of the simulation results, we can see that error(*t*) converges to zero with time increasing. The individual systems achieve complete synchronization because the coupled term vanishes. Some chaotic attractors after synchronization achievement are shown in Fig 4.

## Conclusions

Unlike the Brown process whose almost all sample paths are continuous, the Poisson process is a jump process and has the sample paths which are right-continuous and have left limits. Therefore, it should be pointed out that there is a great difference between the stochastic integral with respect to the Brown process and the one with respect to the Poisson process.

In this paper, we apply the stability theory of SDE driven by Poisson process to study the complete synchronization of the global coupled dynamical network perturbed by different Poisson noises, and sufficient conditions of the complete synchronization with probability 1 are established. Finally, numerical examples are provided to demonstrate the effectiveness of the proposed approach. This paper presents a globally stochastic asymptotic synchronization criterion for complex networks perturbed by the Poisson noise. We conclude that Poisson noise can induce the complete synchronization in the global coupled dynamical network on actual situations.

## Supporting information

S1 Dataset (DAT)
